# The effect of solarium light therapy on selected biological and biochemical parameters of peripheral blood in young and old horses

**DOI:** 10.1371/journal.pone.0304290

**Published:** 2024-05-24

**Authors:** Aleksandra Orzołek, Katarzyna Teresa Rafalska, Anna Domosławska-Wyderska, Agata Monika Rafalska, Anna Dziekońska, Ewa Jastrzębska, Dominika Dobbek

**Affiliations:** 1 Faculty of Animal Bioengineering, Department of Animal Biochemistry and Biotechnology, University of Warmia and Mazury, Olsztyn, Poland; 2 Faculty of Veterinary Medicine, Department of Animal Reproduction with Clinic, University of Warmia and Mazury, Olsztyn, Poland; 3 Faculty of Animal Bioengineering, Department of Horse Breeding and Riding, University of Warmia and Mazury, Olsztyn, Poland; Xijing Hospital, Air Force Medical University, CHINA

## Abstract

The aim of the study was to assess the impact of solarium light therapy on selected biological and biochemical parameters of peripheral blood in recreational horses. The study involved 10 horses divided into two groups of young (aged 5 to 7 years) and old (aged 14 to 19 years) individuals. All animals participated in light therapy sessions every other day. Blood was sampled three times during the study: before the treatment, after five light sessions, and after ten light sessions. Morphological parameters, the activity of antioxidant enzymes, TAS values, and the levels of glutathione (GSH), vitamin D_3_, vitamin C, and malondialdehyde (MDA) were measured in the whole blood. Light therapy contributed to an increase in MCV, HDW, MCVr, CHr and MPV indices, and simultaneously a decrease in the basophil counts, MCHC, RDW and CHCMr indices in both groups of horses (p ≤ 0.05). At the same time reticulocytes fell in older whereas white blood cells and monocytes counts expanded in younger individuals. The treatment also increased the activity of glutathione reductase (GR) and glutathione peroxidase (GPx) in young but decreased the activity of mentioned enzymes in blood plasma of old horses. The total antioxidant status (TAS) of the blood plasma rose progressively, whereas GSH levels declined in all individuals. Moreover, vitamin D_3_ levels did not change, whereas vitamin C levels gradually decreased during the experiment. The therapy also helped to reduce levels of MDA in the blood plasma, especially of older horses (p ≤ 0.05). In turn, GPx and GR activities as well as MDA levels significantly declined, whereas GSH levels notably elevated in erythrocytes (p ≤ 0.05). Solarium light therapy appears to have a beneficial impact on the morphological parameters and antioxidant status of blood in recreational horses in the winter season. However, the observed results could in part be attributed to the natural physiological adaptation of each individual organism to the treatment.

## Introduction

Light therapy involves laser light, light emitting diodes (LED), ultraviolet (UV) light, infrared (IR) light, and tanning beds [1]. A solarium is a phototherapeutic device that is used for both treatment support and health protection. Solarium units emit indoor radiation [2]. Equine solarium units generally rely on IR and UV light. Infrared radiation constitutes the basis of solarium treatment, while UV light emitted by lamps can be used optionally. The therapeutic effects of solarium treatment are determined by the type of radiation as well as wavelength. Ultraviolet radiation, IR light, and blue light are used mainly in rehabilitation [3]. In animals, light therapy is used primarily in the treatment of musculoskeletal injuries, neurological disorders, wounds, and pain [4]. Phototherapy also supports digestive function, accelerates wound healing, boosts the overall immune response, improves respiratory function, and enhances general body performance [5]. Infrared light is a type of electromagnetic radiation that can be applied in the treatment of chronic and subacute inflammatory conditions, neuralgia, certain rheumatic and dermatological conditions, post-traumatic states, and other disorders [6]. In turn, ultraviolet light accelerates metabolism, enhances bone mineralization, stimulates endocrine glands, improves oxygen metabolism, decreases blood pressure, and positively affects the hematopoietic system. Ultraviolet therapy delivers positive results in the treatment of skin diseases (such as psoriasis) [7], selected respiratory disorders, laryngological diseases, rheumatological conditions, convalescence, bone healing, and endocrine gland hypofunction [8].

Previous research has shown that IR radiation stimulates fibroblasts, increases collagen synthesis, and upregulates the expression of transforming growth factor-beta 1 (TGF-β1) in rat wounds [9]. An IR laser was found to enhance cerebral blood flow and elevate nitric oxide levels in mice [10]. Ultraviolet light boosted the immune response by reducing host-virus interactions in COVID-19 patients [11], improved treatment of atopic dermatitis in dogs [12], and even accelerated the healing of skin wounds in iron-deficient rats [13]. However, the effects of UV treatment in horses are more ambiguous. Exposure to UV light has been shown to be an effective and safe treatment for equine corneal ulcers [14], and it significantly increased skin perfusion [15]. However, in a study by Michanek et al. [16], IR phototherapy did not exert a direct effect on wound healing in horses. Walski et al. [17] demonstrated that blood photobiomodulation induced by NIR light reduced oxidative damage during haemodialysis (HD) and that this method can be regarded as a concurrent pre-treatment approach for HD in humans.

Haematological and serum biochemical parameters play a crucial role in diagnosing diseases and predicting prognosis, while also providing insights into an animal’s metabolic status [18]. The variability in blood parameters of horses can be influenced by factors such as breed, physiological state, health condition, housing environment, gender, and age. With aging, horses undergo various physiological changes [19], including alterations in erythrocyte indices [20,21] and the white blood cell system, which is essential for proper immune function [21]. No significant effect of aging was observed on amounts of reactive oxygen metabolites and biological antioxidant potential concentration in horses so far [19]. Irradiation affects the haematological and biochemical parameters of peripheral blood in humans [22] and selected animal species [23,24]. Even though phototherapy is widely applied in physiotherapy, the clinical efficacy of solarium usage in horses has not been investigated to date. Thus, the aim of this study was to investigate the impact of light therapy on selected morphological and biochemical peripheral blood parameters in horses.

## Materials and methods

### Animals

The experiment was conducted on 10 geldings divided into two age groups each: young horses (aged 5–7 years) and old horses (aged 14–19 years). Seven animals were of the Silesian breed and three of the Polish sport horse breeds. The experiment lasted from the end of November to the end of December 2021. The animals were kept in the Equestrian Centre in Kortowo (53,753569°N, 20,455521°E), which constitutes the property of the University of Warmia and Mazury in Olsztyn (UWM). The centre has suitable facilities for maintaining, breeding, and recreational use of horses. The horses were kept under standard zoohygienic conditions. Both before and during the study, the animals were fed a regular diet composed solely of hay and oats. Each day the horses were put into the paddock for a few hours and used recreationally for one hour. Both young and old horses were used recreationally in the same way.

### Light therapy

Light therapy was conducted in the Equestrian Centre of the Department of Horse Breeding and Riding of the UWM. The research was carried out during the winter season when the sunlight angle changes, thus decreasing temperature and the amount of sunlight reaching the Earth’s surface. The timing of the treatments was planned, and weather forecasts were analysed to select days with the lowest expected sun exposure and lowest temperature. Phototherapy was administered using a LEAR horse solarium unit (Lear, Poland). The solarium unit was equipped with 20 lamps emitting infrared light (BR125 IR 250W E27 230-250V Red 1CT/10, Phillips) with a power of 250 W and two lamps emitting UV light (Cleo Compact 20 W, Phillips). Each group of horses participated in a total of 10 solarium treatments that were administered every other day. The duration of each session was 15 minutes, and the same radiation dose was applied in each treatment. The IR radiation treatment was administered during the entire procedure, whereas UV light was applied only in the end of the treatment (up to 2 minutes). During the procedure, horses remained in a standing position, and irradiation was applied mainly to the dorsal part of the body. The animals had a natural hair coat characteristic of the winter period. During each session, the horses wore protective goggles to protect the eyes and enhance the animals’ comfort.

### Peripheral blood collection and sample preparation for analyses

Blood samples for analyses were collected three times i.e. before phototherapy (0 radiations), after five sessions (5 radiations), and after ten sessions (10 radiations). Blood was collected around 7.00 a.m. before the morning feeding. From each horse, approximately16 mL of blood was drawn from the jugular vein. The whole blood was divided into four tubes approximately 4 ml each. Two of them contained the EDTA anticoagulant and one of which was used for morphology with a blood smear test, and another one for the determination of total antioxidant status—TAS, glutathione–GSH content, vitamin C content, and MDA levels. One tube contained heparin served for the determination of the activity of antioxidant enzymes i.e. superoxide dismutase (SOD), glutathione peroxidase (GPx), glutathione reductase (GR) and catalase (CAT). The last sterile tube without preservative was used for the determination of vitamin D_3_ levels. Blood samples were collected by a qualified and licensed veterinarian. The study was approved by the local Ethical Review Board in Olsztyn (approval number 5/2022) under the guidance of the National Ethics Committee for Animal Experimentation in Poland.

The tubes intended for analyses of morphology with a blood smear test and determination of vitamin D_3_ levels were transported to the external research laboratories within one hour of sample collection. The remaining tubes, intended for testing the peripheral blood antioxidant status were transported to the laboratory of the Department of Animal Biochemistry and Biotechnology of the UWM and prepared for further analysis.

And there, firstly, whole blood was centrifuged at 3000 x g for 10 minutes to separate the plasma from morphotic elements. The plasma was then collected into pre-prepared Eppendorf tubes and frozen at -80°C. The separated erythrocytes were washed with 0.9% NaCl solution and centrifuged at 5000 x g for 5 minutes at room temperature to remove residual plasma. The obtained sediment was also frozen at -80°C until further analysis. The plasma was thawed before analysis. The separated erythrocytes were diluted with 0.9% NaCl solution (10 to 30 times, depending on the type of measurement) and then kept at 5°C for 15 minutes to allow the erythrocytes to settle. After this stage, all samples were centrifuged at 10,000 x g for 5 minutes, and biochemical parameters were determined in the obtained supernatants. All biochemical analyses were performed using the BECKMAN COULTER DU 800 spectrophotometer (Beckman Coulter Diagnostics, United States).

### Blood morphology evaluation

Blood morphology and smear tests were performed by experienced laboratory staff of the Department of Internal Diseases with a Clinic of the Faculty of Veterinary Medicine of the UWM in Olsztyn. All measurements were conducted with the use of an ADVIA 2120i haematology analyser (Siemens, Germany) which is a laser-scattering flow cytometry-based system. Determinations were carried out in whole blood samples collected in EDTAK_2_ tubes.

The blood morphology evaluation included:

1. the total count of the white blood cells (WBC) with differential leucocyte counts i.e. neutrophils (NEUT), lymphocytes (LYMPH), monocytes (MONO), eosinophils (EOS), basophils (BASO) and large unstained cells (LUC),
2. the total count of the erythrocytes (RBC), the haemoglobin content (HGB), the haematocrit value (HCT) and indices of red blood cells i.e. the mean corpuscular volume (MCV), the mean corpuscular haemoglobin (MCH), the mean corpuscular haemoglobin concentration (MCHC), the red cell distribution width (RDW), the haemoglobin distribution width (HDW),
3. the total count of reticulocytes (RETIC) and their indices i.e. the mean corpuscular volume of reticulocyte (MCVr), the corpuscular haemoglobin concentration mean of reticulocyte (CHCMr), the mean content haemoglobin of reticulocyte (CHr),
4. the total count of platelets (PLT) and the mean platelet volume (MPV).

### Activity of antioxidant enzymes

The activity of antioxidant enzymes was measured in both the blood plasma and erythrocytes lysed from peripheral blood. Enzyme activity was expressed in international units per millilitre (U/mL) or litre (U/L) or converted to international units per milligram or gram of haemoglobin (U/mg Hb or U/g Hb).

#### Assessment of superoxide dismutase (SOD) activity

Superoxide dismutase activity was measured using the Ransod kit from Randox (Crumlin, United Kingdom) according to the manufacturer’s instructions. This method is based on the ability of the xanthine-xanthine oxidase (XOD) system to generate superoxide anions which react with 2-(4-iodophenyl)-3-(4-nitrophenyl)-5-phenyltetrazolium chloride (INT) to form a red reaction product—formazan. One unit of enzyme activity was defined as the amount of enzyme that leads to 50% inhibition of INT reduction at 37°C and pH 7.0.

#### Assessment of glutathione peroxidase (GPx) activity

Glutathione peroxidase activity was determined using the Ransel kit from Randox (Crumlin, United Kingdom) according to the manufacturer’s protocol. In this method, GPx is used as a catalyst for the oxidation of GSH by cumene hydroperoxide. In the presence of glutathione reductase (GR) and NADPH, oxidised glutathione (GSSG) is immediately converted to its reduced form (GSH), and NADPH is oxidised to NADP+. Absorbance was measured at a wavelength of 340 nm at 37°C.

#### Assessment of glutathione reductase (GR) activity

Glutathione reductase activity was measured using the GLUT-RED kit from Randox (Crumlin, United Kingdom) according to the manufacturer’s instructions. Glutathione reductase catalyses the reduction of glutathione (from GSSG to GSH) in the presence of NADPH which is simultaneously oxidised to NADP+. The decrease in absorbance, measured at 340 nm, is a measure of the enzyme’s activity.

#### Assessment of catalase (CAT) activity

Catalase activity was determined with the use of a Catalase Assay Kit (Merck Life Science, Merck KGaA, Darmstadt, Germany) according to the manufacturer’s instructions. Catalase easily decomposes H_2_O_2_. The remaining H_2_O_2_ reacts with the quinoneimine dye and forms a coupling product which can be measured spectrophotometrically at 520 nm. One unit of CAT decomposes l M H_2_O_2_ per minute at a substrate concentration of 50 mM H_2_O_2_ at 25°C and pH 7.0.

### Assessment of total antioxidant status (TAS)

Total antioxidant capacity (TAC) was measured using a Total Antioxidant Status kit from Randox (Crumlin, United Kingdom). All reagents were prepared according to the manufacturer’s instructions. In the first step, peroxidase catalyses one-electron oxidation of ABTS by hydrogen peroxide which results in the formation of a green ABTS+ radical cation. In this method, ABTS® (2,2’-azinobis(3-ethylbenzothiazoline-6-sulfonic acid)) is incubated with peroxidase (metmyoglobin) and H_2_O_2_, which leads to the formation of the cationic form ABTS®+ and blue-green colouration of the test solution. Colour intensity is measured at a wavelength of 600 nm. The degree to which antioxidants inhibit the formation of a coloured compound is determined by their concentration in the sample.

### Determination of glutathione (GSH) levels

Glutathione levels were measured using the Bioxytech GSH-400 kit (Aoxre, Bio-Sciences, CA, USA) according to the provided protocol. During the test, the patented reagent R1 (4-chloro-1-methyl-7-trifluoromethyl-quinolinium methylsulfate) reacts with mercaptans (RSH) present in the sample to form substitution products (thioethers). Thioethers are converted to chromophoric thione by reagent R2 (30% NaOH). The obtained product has an absorbance maximum at 400 nm at 25°C. Glutathione levels were expressed in μmol/ml for the blood plasma and μmol/g Hb for erythrocytes.

### Determination of vitamins levels

#### Determination of vitamin C level

Vitamin C levels were measured in both the blood plasma and erythrocytes lysed as described by Lowry, Lopez, and Bessey [25]. 200 μl of the analysed sample and 800 μl of 5% tricarboxylic acid (TCA) were placed in a centrifuge tube. The contents were stirred and centrifuged at 14000 x g. The obtained pellet was discarded, and the supernatant was retained for further analysis. Meanwhile, a standard solution (10 mg of ascorbate dissolved in an appropriate volume of 5% TCA) was prepared. In the next step, 200 μl of dilution containing 2 g of 2,4-dinitrophenylhydrazine, 0.25 g of thiosemicarbazide, 0.03 g of CuSO_4_ x 5 H_2_O dissolved in 100 mL of 9 N H_2_SO_4_, were added to 600 μl of the standard or the analysed samples. The tubes were sealed with parafilm and placed in a water bath at 37°C for 4 hours. After incubation, all samples were cooled in an ice bath. 1 mL of cold 65% H_2_SO_4_ was added to each tube, and the contents were thoroughly stirred. The prepared samples were kept at room temperature for 30 minutes. After incubation, absorbance was measured with a spectrophotometer at a wavelength of 520 nm.

The following equation was used to calculate vitamin C levels:

VitC[mg%]=Ab/Asx1

where:

Ab—absorbance of the analysed sample

As—absorbance of the standard solution

#### Determination of vitamin D_3_ level

Serum vitamin D_3_ levels were determined using the eCobas411 device (Roche Diagnostics, Switzerland) in a commercial reference animal laboratory.

#### Measurement of malondialdehyde (MDA) levels

Blood MDA levels were measured using the MDA-586 Assay Kit (Aoxre, Bio-Sciences, CA, USA) according to the manufacturer’s instructions. The assay is based on the reaction of N-methyl-2-phenylindole (R1, NMPI) with MDA at 45°C. One molecule of MDA reacts with 2 molecules of NMPI to yield a stable carbocyanine dye. The concentration of MDA in a sample is determined from the absorbance of the probe and the standard curve at 586 nm in the MDA-586 assay. The results were expressed in μmol MDA/ml for the blood plasma or μmol MDA/g Hb for erythrocytes.

### Statistical analysis

Data were analysed in the Statistica program (version 13.1, StatSoft, Poland). The normality of data distribution was analysed using Shapiro–Wilk test. The data that were not normally distributed were transformed accordingly. Selected blood morphological and biochemical parameters were compared by one-way (age or light therapy) and two-way (age x light therapy) ANOVA. Significant main effects were compared using the Fisher’s LSD (post-hoc) test, and the results were regarded as significantly different at p ≤ 0.05. The results were expressed as means ± SD.

## Results

### The impact of age on selected peripheral blood parameters

Results of ANOVA confirmed that age of horses significantly affected MCV, MCH, RDW, CHr and MCV indices (Table 1). The age was also associated with the activity of GPx, TAS status, GSH and vitamin D_3_ content in blood plasma as well as CAT and GR activities in erythrocytes (Table 2).

**Table 1:** Sources of variation in the morphological parameters of blood of examined horses in ANOVA.

Parameters	Age		Light therapy		Age x Light therapy	
	F-value	p-value	F-value	p-value	F-value	p-value
WBC [10^3^/μl]	0.010	0.922	0.392	0.680	0.935	0.406
NEUT [10^3^/μl]	**6.25**	**0.020**	0.366	0.698	0.596	0.559
LYMPH [10^3^/μl]	**11.51**	**0.002**	0.019	0.980	0.121	0.887
MONO [10^3^/μl]	0.094	0.762	0.472	0.630	1.02	0.376
EOS [10^3^/μl]	0.003	0.955	0.100	0.905	0.001	0.999
BASO [10^3^/μl]	0.409	0.528	1.636	0.216	1.64	0.216
LUC [10^3^/μl]	3.06	0.093	0.404	0.672	0.064	0.938
RBC [10^6^/μl]	0.211	0.650	0.244	0.785	0.022	0.979
**Hb [g/l]**	4.23	0.051	0.185	0.833	0.142	0.869
**HCT [%]**	2.02	0.168	0.817	0.454	0.052	0.949
**MCV [fl]**	**6.69**	**0.014**	**3.590**	**0.043**	0.003	0.997
**MCH [pg]**	**9.90**	**0.004**	0.102	0.903	0.033	0.967
**MCHC [g/dl]**	3.55	0.072	**21.21**	**0.000**	0.178	0.838
**RDW [%]**	**23.56**	**0.000**	**18.53**	**0.000**	0.076	0.927
**HDW [g/dl]**	1.57	0.223	**46.68**	**0.000**	0.497	0.614
RETIC [10^6^/μl]	3.15	0.089	2.21	0.132	0.286	0.754
**MCVr [fl]**	1.69	0.206	**7.42**	**0.003**	0.173	0.842
**CHCMr [g/dl]**	3.94	0.059	**3.67**	**0.041**	0.410	0.668
**CHr [pg]**	**5.35**	**0.030**	**5.52**	**0.011**	0.230	0.796
PLT [10^3^/μl]	0.528	0.475	0.162	0.852	0.540	0.590
**MPV [fl]**	**14.00**	**0.001**	**11.18**	**0.000**	0.483	0.623

F-value—the ratio of between group variation and within group variation.

Values given **in bold** are statistically significant (p ≤ 0.05).

**WBC**–white blood cells, **NEUT**–neutrophiles, **LYMPH**–lymphocyte, **MONO**–monocytes, **EOS**–eosinophils, **BASO**–basophils, **LUC**—large unstained cells, **RBC**–red blood cells, **Hb**–haemoglobin, **HCT**–haematocrit, **MCV**—mean corpuscular volume, **MCH**—mean corpuscular haemoglobin, **MCHC**—mean corpuscular haemoglobin concentration, **RDW**—red cell distribution width, **HDW**—haemoglobin distribution width, **RETIC**—total count of reticulocytes, **MCVr**—mean corpuscular volume of reticulocyte, **CHCMr**—corpuscular haemoglobin concentration mean of reticulocyte, **CHr**—mean content haemoglobin of reticulocyte, **PLT**—total count of platelets, **MPV**—mean platelet volume.

**Table 2:** Sources of variation in the parameters of antioxidant status of blood of examined horses in ANOVA.

Parameters		Age		Light therapy		Age x Light therapy	
		F-value	p-value	F-value	p-value	F-value	p-value
**Blood plasma**	**SOD [U/ml]**	0.128	0.724	0.160	0.853	0.049	0.952
	**GPx [U/l]**	**7.38**	**0.012**	1.44	0.256	3.10	0.064
	**GR [U/l]**	0.002	0.963	0.115	0.892	**7.29**	**0.003**
	**CAT [U/ml]**	1.60	0.218	**3.51**	**0.046**	0.093	0.911
	**TAS [mmol/l]**	**15.84**	**0.000**	**46.47**	**0.000**	**11.05**	**0.000**
	**GSH [μM/ml]**	**4.67**	**0.041**	1.57	0.228	0.902	0.419
	**Vitamin D**_**3**_ **[ng/ml]**	**6.34**	**0.019**	0.224	0.801	0.957	0.398
	**Vitamin C [mg%]**	3.85	0.062	**4.07**	**0.030**	1.44	0.257
	**MDA [μM/ml]**	0.99	0.330	0.681	0.516	2.53	0.100
**Erythrocytes**	**SOD [U/mg Hb]**	0.838	0.369	0.348	0.709	0.131	0.878
	**GPx [U/g Hb]**	1.16	0.293	**32.14**	**0.000**	**4.66**	**0.020**
	**GR [U/g Hb]**	**6.03**	**0.022**	**33.57**	**0.000**	**6.81**	**0.005**
	**CAT [U/g Hb]**	**4.73**	**0.040**	0.010	0.990	0.150	0.861
	**TAS [mmol/l]**	0.002	0.966	**44.54**	**0.000**	0.571	0.572
	**GSH [μM/g Hb]**	0.101	0.753	**80.51**	**0.000**	0.081	0.922
	**Vitamin C [mg%]**	0.566	0.459	0.030	0.971	1.70	0.203
	**MDA [μM/g Hb]**	3.36	0.079	**6.07**	**0.007**	1.03	0.372

F-value–the ratio of between group variation and within group variation.

Values given **in bold** are statistically significant (p ≤ 0.05).

**SOD**—superoxide dismutase, **GPx**—glutathione peroxidase, **GR**—glutathione reductase, **CAT**–catalase, **TAS**—total antioxidant status, **GSH**–glutathione, **MDA**–malondialdehyde.

Lymphocyte (LYMPH) counts and red cell distribution width (RDW) were significantly higher in younger horses. In turn, neutrophil (NEUT) counts, Hb content and indices such as mean corpuscular volume (MCV), mean corpuscular haemoglobin (MCH), mean content haemoglobin of reticulocyte (CHr) and mean platelet volume (MPV) were higher (p ≤ 0.05) in older horses (Table 3).

**Table 3:** Comparison of morphological parameters in young and old horses.

Parameters	Younger	Older	p-value
	Mean ± SD	Mean ± SD	
WBC [10^3^/μl]	5.34 ± 0.82	5.68 ± 0.60	0.92
NEUT [10^3^/μl]	**2.61 ± 0.27**	**3.42 ± 0.60**	**0.015**
LYMPH [10^3^/μl]	**2.28 ± 0.2**	**1.82 ± 0.35**	**0.001**
MONO [10^3^/μl]	0.28 ± 0.05	0.26 ± 0.13	0.76
EOS [10^3^/μl]	0.10 ± 0.04	0.10 ± 0.09	0.95
BASO [10^3^/μl]	0.03 ± 0.01	0.04 ± 0.01	0.55
LUC [10^3^/μl]	0.04 ± 0.01	0.03 ± 0.02	0.074
RBC [10^6^/μl]	7.87 ± 0.38	7.80 ± 0.95	0.63
**Hb [g/l]**	**128.2 ± 5.76**	**134.3 ± 9.04**	**0.037**
**HCT [%]**	38.40 ± 1.80	39.96 ± 3.45	0.15
**MCV [fl]**	**48.86 ± 2.38**	**51.42 ± 3.05**	**0.018**
**MCH [pg]**	**16.30 ± 0.64**	**17.50 ± 1.07**	**0.002**
**MCHC [g/dl]**	33.42 ± 0.53	34.00 ± 0.33	0.23
**RDW [%]**	**20.08 ± 0.41**	**19.26 ± 0.44**	**0.003**
**HDW [g/dl]**	2.79 ± 0.15	2.71 ± 0.16	0.55
RETIC [10^6^/μl]	25.30 ± 13.10	21.10 ± 23.05	0.09
**MCVr [fl]**	51.38 ± 3.10	54.32 ± 3.92	0.28
**CHCMr [g/dl]**	33.74 ± 0.23	34.00 ± 1.12	0.07
**CHr [pg]**	**16.98 ± 0.97**	**18.08 ± 0.97**	**0.05**
PLT [10^3^/μl]	142.6 ± 39.43	109.6 ± 33.20	0.19
**MPV [fl]**	**6.48 ± 0.20**	**6.86 ± 0.26**	**0.008**

Values given **in bold** are statistically significant (p ≤ 0.05).

**WBC**–white blood cells, **NEUT**–neutrophiles, **LYMPH**–lymphocyte, **MONO**–monocytes, **EOS**–eosinophils, **BASO**–basophils, **LUC**—large unstained cells, **RBC**–red blood cells, **Hb**–haemoglobin, **HCT**–haematocrit, **MCV**—mean corpuscular volume, **MCH**—mean corpuscular haemoglobin, **MCHC**—mean corpuscular haemoglobin concentration, **RDW**—red cell distribution width, **HDW**—haemoglobin distribution width, **RETIC**—total count of reticulocytes, **MCVr**—mean corpuscular volume of reticulocyte, **CHCMr**—corpuscular haemoglobin concentration mean of reticulocyte, **CHr**—mean content haemoglobin of reticulocyte, **PLT**—total count of platelets, **MPV**—mean platelet volume.

Glutathione peroxidase (GPx) activity was substantially higher in older horses. Despite blood plasma glutathione reductase (GR) activity was similar in both age groups, GR activity in erythrocytes was more than twice higher in younger than in older horses (p ≤ 0.05). Glutathione content in blood plasma was significantly higher in younger horses, whereas GSH level in erythrocytes was comparable between both age groups. The content of vitamin C in blood plasma was higher in younger individuals (p ≤ 0.05), whereas malondialdehyde (MDA) level was lower in older animals (p ≤ 0.05) (Table 4).

**Table 4:** Comparison of parameters of antioxidant status of blood in young and old horses.

	Parameters	Younger	Older	p-value
		Mean ± SD	Mean ± SD	
**Blood plasma**	**SOD [U/ml]**	48.16 ± 14.24	45.96 ± 1.69	0.68
	**GPx [U/l]**	**901.1 ± 94.86**	**1527.9 ± 132.4**	**0.018**
	**GR [U/l]**	270.7 ± 27.60	301.8 ± 45.34	0.97
	**CAT [U/ml]**	66.13 ± 12.60	82.56 ± 32.45	0.24
	**TAS [mmol/l]**	1.61 ± 0.09	1.46 ± 0.10	0.08
	**GSH [μM/ml]**	**807.3 ± 88.60**	**559.2 ± 30.94**	**0.04**
	**Vitamin D**_**3**_ **[ng/ml]**	7.33 ± 0.34	7.75 ± 0.97	0.7
	**Vitamin C [mg%]**	**21.57 ± 9.79**	**6.50 ± 2.92**	**0.045**
	**MDA [μM/ml]**	**378.5 ± 53.74**	**159.3 ± 50.65**	**0.03**
**Erythrocytes**	**SOD [U/mg Hb]**	1.32 ± 0.02	1.27 ± 0.06	0.34
	**GPx [U/g Hb]**	31.46 ± 0.78	28.03 ± 1.98	0.57
	**GR [U/g Hb]**	**18.28 ± 2.87**	**8.32 ± 2.31**	**0.02**
	**CAT [U/g Hb]**	**61.03 ± 1.15**	**57.79 ± 2.08**	**0.027**
	**TAS [mmol/l]**	63.19 ± 15.81	53.44 ± 4.67	0.98
	**GSH [μM/g Hb]**	108.5 ± 18.23	91.46 ± 28.16	0.9
	**Vitamin C [mg%]**	0.15 ± 0.04	0.12 ± 0.06	0.45
	**MDA [μM/g Hb]**	31.09 ± 9.16	17.64 ± 1.90	0.12

Values given **in bold** are statistically significant (p ≤ 0.05).

**SOD**—superoxide dismutase, **GPx**—glutathione peroxidase, **GR**—glutathione reductase, **CAT**–catalase, **TAS**—total antioxidant status, **GSH**–glutathione, **MDA**–malondialdehyde.

### The impact of light therapy on selected peripheral blood parameters

Analysis of variance results revealed that the light therapy affected mainly morphological indices of blood cells i.e. MCV, MCHC, RDW, HDW, MCVr, CHCMr, CHr and MPV (Table 1). Furthermore, it was found that light therapy influenced the activity of CAT, TAS value and vitamin C content in blood plasma. But the most significant effect was observed in erythrocytes of both examined age groups and it was demonstrated through activities of GPx and GR, TAS value, GSH content and MDA level (Table 2).

Subsequent sessions of light therapy decreased (p ≤ 0.05) basophile counts, the mean corpuscular haemoglobin concentration (MCHC), the mean haemoglobin concentration in reticulocytes (CHCMr), and RDW indices. In the long run, applied treatments notably increased the values of MCV, HDW, MCVr, CHr, and MPV. It should be noted that in older horses reticulocyte counts have dropped significantly as soon as after five light therapy sessions (Table 5).

**Table 5:** Morphological parameters in young and old horses before (0) and after five (5) and ten (10) light therapy sessions.

Parameters	Young			Old		
	0	5	10	0	5	10
	Mean ± SD	Mean ± SD	Mean ± SD	Mean ± SD	Mean ± SD	Mean ± SD
WBC [10^3^/μl]	5.34^**b**^ ± 0.82	6.00^**b**^ ± 0.72	6.42^**a**^ ± 0.48	5.68 ± 0.60	5.52 ± 0.61	5.63 ± 0.97
NEUT [10^3^/μl]	2.61 ± 0.27	3.14 ± 0.96	2.64 ± 0.57	3.42 ± 0.60	3.37 ± 0.64	3.45 ± 0.80
LYMPH [10^3^/μl]	2.28 ± 0.72	2.38 ± 0.62	2.35 ± 0.41	1.82 ± 0.35	1.75 ± 0.16	1.70 ± 0.29
MONO [10^3^/μl]	0.28^**b**^ ± 0.05	0.28^**b**^ ± 0.04	0.31^**a**^ ± 0.04	0.26 ± 0.13	0.25 ± 0.05	0.33 ± 0.13
EOS [10^3^/μl]	0.10 ± 0.04	0.10 ± 0.04	0.09 ± 0.03	0.10 ± 0.09	0.10 ± 0.08	0.09 ± 0.07
BASO [10^3^/μl]	0.03^a^ ± 0.01	0.03^**a**^ ± 0.07	0.02^**b**^ ± 0.01	0.04^**a**^ ± 0.01	0.03^**a**^ ± 0.01	0.02^**b**^ ± 0.01
LUC [10^3^/μl]	0.04 ± 0.01	0.03 ± 0.01	0.04 ± 0.01	0.03 ± 0.02	0.03 ± 0.01	0.03 ± 0.02
RBC [10^6^/μl]	7.87 ± 0.38	7.88 ± 0.35	7.67 ± 0.38	7.80 ± 0.95	7.69 ± 0.70	7.58 ± 0.98
**Hb [g/l]**	128.2 ± 5.22	129.6 ± 6.47	126.8 ± 6.50	134.3 ± 9.04	133.6 ± 5.27	133.2 ± 11.65
**HCT [%]**	38.40 ± 1.80	39.66 ± 1.56	39.86 ± 1.70	39.96 ± 3.45	40.56 ±2.03	41.38 ± 3.81
**MCV [fl]**	48.86^**b**^ ± 2.38	50.38^**b**^ ± 2.87	52.02^**a**^ ± 2.54	51.42^**b**^ ± 3.05	52.92^**b**^ ± 2.37	54.74^**a**^ ± 2.96
**MCH [pg]**	16.30 ± 0.64	16.48 ± 0.81	16.54 ± 0.73	17.50 ± 1.07	17.46 ± 1.14	17.64 ± 1.19
**MCHC [g/dl]**	33.42^**a**^ ± 0.53	32.70^**b**^ ± 0.80	31.76^**c**^ ± 0.51	34.00^**a**^ ± 0.33	32.96^**b**^ ± 0.72	32.16^**c**^ ± 0.61
**RDW [%]**	20.08^**a**^ ± 0.41	18.84^**b**^ ± 0.68	19.14^**b**^ ± 0.38	19.26^**a**^ ± 0.44	18.06^**b**^ ± 0.43	18.20^**b**^ ± 0.46
**HDW [g/dl]**	2.79^**b**^ ± 0.15	2.48^**c**^ ± 0.39	3.27^**a**^ ± 0.18	2.71^**b**^ ± 0.16	2.30^**c**^ ± 0.06	3.26^**a**^ ± 0.08
RETIC [10^6^/μl]	25.30 ± 13.10	20.16 ± 21.55	14.84 ± 17.04	21.10^**a**^ ± 23.05	5.82^**b**^ ± 6.29	2.64^**b**^ ± 1.58
**MCVr [fl]**	51.38^**b**^ ± 3.10	53.36^**b**^ ± 2.82	60.20^**a**^ ± 6.35	54.32^**b**^ ± 3.92	56.28^**b**^ ± 3.74	61.00^**a**^ ± 6.63
**CHCMr [g/dl]**	33.74^**a**^ ± 0.23	33.64^**a**^ ± 0.59	32.52^**b**^ ± 0.93	34.00^**b**^ ± 1.12	34.64^**a**^ ± 1.10	33.44^**b**^ ± 1.51
**CHr [pg]**	16.98^**b**^ ± 0.97	17.58^**b**^ ± 0.91	19.12^**a**^ ± 1.69	18.08^**b**^ ± 0.97	19.10^**b**^ ± 1.25	19.84^**a**^ ± 1.81
PLT [10^3^/μl]	142.6 ± 39.43	120.6 ± 67.36	140.4 ± 35.14	109.6 ± 33.20	128.8 ± 35.67	129.8 ± 46.26
**MPV [fl]**	6.48^**b**^ ± 0.20	6.72^**b**^ ± 0.44	7.06^**a**^ ± 0.42	6.86^**b**^ ± 0.26	7.10^**b**^ ± 0.35	7.70^**a**^ ± 0.31

Different superscripts “^**a**^, ^**b**^” show significant (p ≤ 0.05) differences in morphological parameters between young and old horses before and after light therapy sessions.

**WBC**–white blood cells, **NEUT**–neutrophiles, **LYMPH**–lymphocyte, **MONO**–monocytes, **EOS**–eosinophils, **BASO**–basophils, **LUC**—large unstained cells, **RBC**–red blood cells, **Hb**–haemoglobin, **HCT**–haematocrit, **MCV**—mean corpuscular volume, **MCH**—mean corpuscular haemoglobin, **MCHC**—mean corpuscular haemoglobin concentration, **RDW**—red cell distribution width, **HDW**—haemoglobin distribution width, **RETIC**—total count of reticulocytes, **MCVr**—mean corpuscular volume of reticulocyte, **CHCMr**—corpuscular haemoglobin concentration mean of reticulocyte, **CHr**—mean content haemoglobin of reticulocyte, **PLT**—total count of platelets, **MPV**—mean platelet volume.

Moreover, light therapy significantly decreased CAT and increased GPx and GR activities in blood plasma of younger animals. TAS parameter gradually rose (p ≤ 0.05) during light therapy application between the two distinguished groups. It should be emphasised that no statistically significant differences in D3 levels were found between the two age groups studied. However, they were indicated for plasma vitamin C concentration. Significant decrease in GPx and GR activity, accompanied together by an increase in TAS parameter, GSH level and a decrease in MDA levels was observed (Table 6).

**Table 6:** Parameters of antioxidant status in young and old horses before (0) and after five (5) and ten (10) light therapy sessions.

Parameters		Young			Old		
		0	5	10	0	5	10
		Mean ± SD	Mean ± SD	Mean ± SD	Mean ± SD	Mean ± SD	Mean ± SD
**Blood plasma**	**SOD [U/ml]**	48.16 ± 14.24	47.52 ± 2.57	38.84 ± 2.71	45.96 ± 1.69	37.28 ± 9.89	37.52 ± 34.14
	**GPx [U/l]**	901.1^**b**^ ± 94.86	1183.2^**ab**^ ± 78.70	1480.2^**a**^ ± 168.3	1527.9 ± 132.4	1798.5 ± 271.9	1368.8 ± 195.3
	**GR [U/l]**	270.7^**b**^ ± 27.60	234.2^**b**^ ± 22.89	336.5^**a**^ ± 7.85	301.8^**ab**^ ± 45.34	328.7^**a**^ ± 35.42	207.5^**b**^ ± 28.32
	**CAT [U/ml]**	66.13^**a**^ ± 12.60	18.56^**b**^ ± 7.82	44.80^**ab**^ ± 8.06	82.56 ± 32.45	42.99 ± 11.61	55.04 ± 12.61
	**TAS [mmol/l]**	1.61^**b**^ ± 0.09	0.98^**c**^ ± 0.15	2.02^**a**^ ± 0.05	1.46^**b**^ ± 0.10	1.69^**b**^ ± 0.08	2.35^**a**^ ± 0.03
	**GSH [μM/ml]**	807.3 ± 88.60	716.1 ± 94.44	642.4 ± 92.20	559.2 ± 30.94	684.5 ± 95.13	495.1 ± 61.45
	**Vitamin D**_**3**_ **[ng/ml]**	7.33 ± 0.34	6.42 ± 0.53	6.36 ± 0.39	7.75 ± 0.97	8.78 ± 1.01	7.97 ± 0.72
	**Vitamin C [mg%]**	21.57^**a**^ ± 9.79	6.49^**ab**^ ± 1.39	3.48^**b**^ ± 1.26	6.50 ± 2.92	2.62 ± 0.94	1.93 ± 0.46
	**MDA [μM/ml]**	378.5 ± 53.74	270.4 ± 89.25	250.4 ± 79.64	159.3^**b**^ ± 50.65	356.4^**a**^ ± 68.59	217.3^**ab**^ ± 59.50
**Erythrocytes**	**SOD [U/mg Hb]**	1.32 ± 0.02	1.29 ± 0.03	1.26 ± 0.07	1.27 ± 0.06	1.22 ± 0.05	1.25 ± 0.06
	**GPx [U/g Hb]**	31.46^**a**^ ± 0.78	16.21^**c**^ ± 1.44	19.98^**b**^ ± 1.34	28.03^**a**^ ± 1.98	21.50^**b**^ ± 1.78	21.92^**b**^ ± 0.95
	**GR [U/g Hb]**	18.28^**a**^ ± 2.87	1.92^**b**^ ± 0.42	1.92^**b**^ ± 0.35	8.32^**a**^ ± 2.31	1.81^**b**^ ± 0.27	2.42^**b**^ ± 1.12
	**CAT [U/g Hb]**	61.03 ± 1.15	60.18 ± 1.52	60.90 ± 1.44	57.79 ± 2.08	58.36 ± 1.24	57.48 ± 1.90
	**TAS [mmol/l]**	63.19^**b**^ ± 15.81	117.8^**a**^ ± 7.87	119.9^**a**^ ± 3.35	53.44^**b**^ ± 4.67	122.3^**a**^ ± 2.04	124.4^**a**^ ± 2.27
	**GSH [μM/g Hb]**	108.5^**b**^ ± 18.23	82.50^**b**^ ± 14.05	395.6^**a**^ ± 36.13	91.46^**b**^ ± 28.16	72.36^**b**^ ± 22.32	400.8^**a**^ ± 40.64
	**Vitamin C [mg%]**	0.11 ± 0.04	0.16 ± 0.04	0.20 ± 0.05	0.16 ± 0.06	0.13 ± 0.03	0.09 ± 0.03
	**MDA [μM/g Hb]**	31.09^**a**^ ± 9.16	13.01^**b**^ ± 2.35	12.81^**b**^ ± 3.66	17.64^**a**^ ± 1.90	10.84^**b**^ ± 1.72	9.22^**b**^ ± 0.76

Different superscripts “^**a**^, ^**b**^” show significant (p≤0.05) differences in parameters of antioxidant status of blood between young and old horses before and after light therapy sessions.

**SOD**—superoxide dismutase, **GPx**—glutathione peroxidase, **GR**—glutathione reductase, **CAT**–catalase, **TAS**—total antioxidant status, **GSH**–glutathione, **MDA**–malondialdehyde.

### The impact of age and light therapy on selected peripheral blood parameters

Two factors did not affect the morphological parameters of peripheral blood (Table 1), but they influenced both glutathione reductase activity and TAS value in blood plasma as well as GPx and GR activities in erythrocytes (Table 2).

## Discussion

Light therapy involves the transmission of light particles known as photons to body tissues. These processes enhance electron transport, enzyme activity, and ATP production, which are the critical determinants of cell growth, tissue repair, and regeneration [26]. Various light-emitting sources and protocols are employed in practice, and treatment parameters such as wavelength, irradiated area, intensity levels, and duration are modified to achieve the desired therapeutic effect [1].

Blood cell counts and chemical components should be monitored to keep horse in a good condition. The results of blood screening tests are a good barometer of overall health [27]. The maintenance of homeostasis plays a fundamental role in the regulation of blood viscosity and blood flow in the circulatory system [28].

### The impact of age on selected peripheral blood parameters in horses

In horses, RBC, WBC, and PLT values decrease with age, which points to a decline in the bone marrow response [21]. Aging of horses’ manifests, among others, by a decrease in lymphocyte (LYMPH) count [29]. Similar observation was made by Czech et al. [30] who noticed that lymphocyte count (LYM) was significantly lower in older individuals. But they also demonstrated that in older horses (aged 10–16 years), white blood cell count (WBC), neutrophil count (NEU), and monocyte count (MID) were significantly higher compared to other age groups. Present study confirmed the part of mentioned observations. Lymphocyte count was significantly higher in young, whereas neutrophil count in old horses. The number of white blood cells slightly increased in older animals, but in non-significant way. Contrary, the monocyte counts were similar among both examined age groups. The counts of degenerated neutrophils are typically lower in horses older than 9 years [31]. However, lower counts of normal neutrophils in young horses in our study may be attributed to the fact that their WBCs have not yet achieved full maturity. Lymphocyte counts are usually higher in younger than in older horses, and the decrease in lymphocyte counts in older horses is associated with the gradual age-related decline in immunocompetence [32]. In horses, physiological leucocytosis can be triggered by excitement or stress [33]. Psychological factors, such as stress during blood collection, cannot be ruled out. According to the age, platelet numbers in horses do not change during the first year of life but age determines a progressive decrease [34]. Contrary, Kisadere et al. [35] demonstrated that PLT in older horses were extremely higher than those that aged 1–5 years. Our results showed that platelet counts were actually higher in younger animals, but the differences between groups were not significant. However, such an outcome might have been caused by the age of geldings, from whom the youngest were five years old. Chikhaoui et al. [36] reported no significant differences in the values of RBC, Ht, Hb, MCV, and MCHC values between age-differentiated groups of horses. Our study confirmed this observation.

The antioxidant defence system is weakened with age [37]. In the blood serum of horses, aged 2–6 years, significantly higher superoxide dismutase (SOD) activity was noted as compared to older ones [38]. Results obtained by Kirschvink et al. [39] showed that age-related changes are most prominent for superoxide dismutase (SOD), which is significantly lower in horses aged over 6 years old. However present study did not validate this remark, as no relationship were found between age and SOD activity in the blood of animals. It is in accordance with previous work performed by Żak et al [19]. On the other hand, we observed that age was associated with the GPx activity in older and GR activity in younger horses. Gondim et al. [40] found that high GR capacity and/or low TBAR concentration improve performance in horses; therefore, higher GR activity in erythrocytes of younger animals from our study could be indicative of their greater endurance and improved circulatory capacity. Moreover, antioxidant potential of blood might have been supported by the presence of low molecular weight antioxidants. In our study, the vitamin C levels were significantly higher in blood plasma of younger horses. Similar observations were noticed by Ralston et al. [41] who reported lower plasma vitamin C concentration in older than in younger horses fed the same diet and housed under the same conditions. We suppose that the higher content of vitamin C in young animals protects their blood against oxidative stress as the other indicators are subjected to quite variable fluctuations. Previous research has shown that MDA levels are higher in older than in younger horses, which indicates that oxidative processes intensify with age. The content of MDA was significantly higher in the blood of animals aged over 10 years old compared to younger individuals [38]. However, present study showed that MDA levels were higher in the blood of young horses. To our minds, this observation can be attributed to the greater instability of the immune system of young individuals or its stronger responsiveness to any stressor.

### The impact of phototherapy on selected peripheral blood parameters in horses

Direct photobiomodulation therapy increases membrane fluidity and membrane potential in erythrocytes, thus reducing their agglutination. Aouthmany [42] found that phototherapy increased Hb degradation in preterm infants. This observation is not fully consistent with the results of the present study, where RBC counts and Hb levels remained fairly stable during light therapy. A marginal and non-significant increase was observed only in HCT values. In a study of newborns, phototherapy decreased the MCV and RDW of erythrocytes, but induced a significant increase in MCHC and monocyte counts [43]. However, in the current study, MCV and RDW values increased, whereas MCHC decreased after phototherapy, which could be attributed to differences in the evaluated species and the specific physiology of equine erythrocytes. In dogs, a positive association was reported between RDW values and biomarkers of oxidative stress [44]. Thus, in the current study, the initial decrease, followed by an increase in RDW values, could point to changes in the antioxidant potential of equine erythrocytes. In horses, *de novo* production of RBCs is associated with greater variability in RDW and, in the majority of cases, with an increase in MCV [45]. A similar trend was noted in this study, but no differences in RBC counts were observed, which could be attributed to delayed erythropoiesis or minor haemolysis of RBCs. Reticulocyte counts decreased gradually due to their maturation or damage. In humans, reticulocytes mature within one day after being released into peripheral blood. In horses, the exact lifespan of reticulocytes is not specified. High reticulocyte counts can increase MCV [46], but an opposite effect was noted in the present study. Moreover, mature reticulocytes are stable and deformable, while mechanical stability and membrane deformability are decreased in younger cells [47]. Therefore, reticulocyte maturation could have influenced reticulocyte counts in the present study. Reticulocyte haemoglobin content is an indicator of effective Hb synthesis [48]. The reticulocyte count is a valuable parameter for quantifying erythropoiesis, but Hb levels in reticulocytes provide additional information about the quality of erythropoiesis and the degree of haemoglobinisation [49]. In the present study, elevated CHr value could be indicative of steady-state erythropoiesis. Light therapy induces a significant (p ≤ 0.05) increase in eosinophil and basophil counts, and a decrease in leukocyte and neutrophil counts. However, monocyte and lymphocyte counts are not affected by the treatment [50]. On the contrary, our study showed a slight decrease in basophil and reticulocyte counts, as well as an increase of total monocytes. Simultaneously, lymphocyte and neutrophil counts stayed rather unaffected. According to research, lymphocyte and neutrophil counts remain stable in horses housed in a paddock during the winter season. Cold environment stabilises mentioned indices without affecting the overall cell count because blood cells are already in equilibrium [51]. The present study was conducted in winter; therefore, main blood parameters were probably stabilised. Drohomirecka et al. [24] observed that low-level light therapy could be a simple and safe method for treating coagulation disorders by preventing PLT destruction and destabilization during extracorporeal circulation. Moreover, Yang et al. [52] demonstrated that low-level light therapy is effective in the treatment of immune thrombocytopenia because it increases total PLT counts. In contrast, Khera and Gupta [53] and Shah et al. [54] reported that phototherapy decreased PLT counts. In the present study we observed steady increase of PLT, however gradual and non-significant. These discrepancies could be attributed to differences in the type and source of light, exposure times, the animals’ health status and, most importantly, the examined species.

Phototherapy can affect the antioxidant potential of cells [55]. Phototherapy increase (p ≤ 0.05) the activity of SOD [56] and GPx [57] in the blood of newborns, but it has no effect on GR activity [58]. According to Ayyappan et al. [57], higher SOD and GPx activity could be attributed to enhanced generation of ROS during phototherapy, as well as enhanced capacity of the antioxidant defence system. However, in our experiment, SOD activity did not increase in response to light therapy. Significant increase was observed in GPx and GR activities but only in young horses. It appears that SOD did not contribute to a reduction in plasma ROS levels and its role has been taken over by increased activities of GPx and GR, especially in younger individuals. In turn, CAT activity initially declined and then began to rise so it may also have contributed to the slow increase in the antioxidant potential of the fluid. Because the antioxidant capacity of the blood plasma simultaneously increased, we deduced that other enzymes or mechanisms were involved. In the present study, phototherapy had a significant impact on GPx and GR activity in erythrocytes, which could be associated with increased production and regeneration of innate antioxidants in RBCs. In the work of Waggiallaah and Alzohairy [59], a decrease in GPx and GR activity was accompanied by a simultaneous decline in GSH levels in erythrocytes, leading to oxidative stress. However, this observation is not fully corroborated by the results of this study. Yes, the decrease in the plasma levels of GPx and GR was accompanied by a decrease in GSH concentration, especially in older animals. However, in erythrocytes GPx and GR activity decreased, whereas GSH levels significantly increased. Therefore, it cannot be postulated that the decline in GPx and GR activity was associated with oxidative stress in erythrocytes. A more likely explanation is that GPx and GR activity in erythrocytes decreased due to an increase in intracellular GSH reserves or more efficient operation of the glutathione regeneration system. The observed increase in TAS seems to undermine the above hypothesis. In the present study, the TAS of younger horses decreased only after first five treatments, but it continued to increase during the entire experiment in older animals. A similar trend was observed by Walski et al. [60] who reported a significant initial decrease, followed by a gradual increase in TAC after 6 h of light therapy in pigs. In younger animals, the initial decrease in TAS can be explained by a potentially greater response to the treatment at the beginning of the study. Better antioxidant capacity of the blood was also confirmed by the GSH content. Despite a non-significant decrease in plasma GSH level also its considerable increase in erythrocytes was observed. IR-A radiation substantially alters the balance between GSH and GSSG in cells and shifts the balance towards oxidation, which leads to an increase in the cellular redox potential. The above can be attributed to increased ROS generation by mitochondria [61]. Both GSSG and glutathione conjugates (GS-X) are actively exported from RBCs when their intracellular concentration is high [62]. This *de novo* re-synthesis may balance the loss of GSH caused by GSSG and GS-X export, and it is regulated by a feedback mechanism [63]. However, little is known about the release of GSH from RBCs into the extracellular pool and its role in homeostasis maintenance. Nevertheless, we observed such a mechanism in the blood of horses as GSH content in plasma rose after previous increase of glutathione in erythrocytes. The greatest advantage of UV phototherapy is that it initiates vitamin D_3_ synthesis, strengthens the body’s defence mechanisms and enhances immunomodulatory effects. The primary physiological need for vitamin D_3_ is typically met through the conversion of 7-dehydrocholesterol (7-DHC) in the skin [64]. However, Azarpeykan et al. [65] demonstrated that horses produce negligible quantities of vitamin D_3_ in the skin after exposure to UVB light and may, therefore, rely on their diet as a primary source of this vitamin. The plasma levels of both 25OHD_2_ and 25OHD_3_ indicate that the vitamin D status of horses is typically low. In horses, these parameters are consistently low, often below 10 ng/ml, regardless of season or geographical latitude [66]. Mäenpää et al. [67] observed consistently low levels of 25OHDx in horses, with minimal seasonal fluctuations. The plasma concentration of 25OHDx was determined at 4.20 ± 0.34 ng/mL in January and at 6.20 ± 0.36 ng/mL in June. In the present study, no differences were observed in vitamin D_3_ levels, which could suggest that the amount of vitamin D_3_ synthesised in the skin was not sufficient to affect the total vitamin D levels in the blood serum of horses. It confirms results obtained by Azarpeykan et al. [65]. There is an ongoing debate on whether animals with hair coats, such as horses and cattle can synthesise vitamin D_3_ in their skin. Some researchers have postulated that a hair coat effectively blocks sunlight from reaching the skin [68]. No change in vitamin D_3_ levels, in present study, could be explained by the fact that the duration of the UV treatment was too short (approximately 2 minutes) or that the applied wavelength was insufficient to stimulate vitamin D_3_ synthesis. Coat colour (95% of the studied animals were bay and chestnut horses) or coat length are also possible explanations. On the other hand, light therapy affected the vitamin C concentration in the blood of the examined animals. Vitamin C levels gradually decreased in both the blood plasma and erythrocytes. Ankur et al. [69] and Hargreaves et. al. [70] demonstrated that vitamin C levels in horses decreased after strenuous exercise or physically challenging race. In the cited studies, phototherapy was often preceded by a typical recreational use; therefore, the decline in vitamin C levels could have been partly associated with physical effort. But more likely explanation is that vitamin C, the main antioxidant in the blood plasma, was depleted because it protected cells from oxidative stress throughout the treatment.

Light therapy should involve low to moderate doses of IR-A radiation to prevent skin damage and photoaging [71]. A study performed by Schroeder et al. [61] revealed that IR-A radiation (760–1440 nm), unlike UV radiation, triggers a retrograde signalling response in normal human skin fibroblasts. Mitochondrial retrograde signalling has been characterised as both a cellular stress [72] and an adaptive response [73]. In the present study, the effect of light treatments became apparent only after several sessions. Light therapy influenced not only blood cells indices, but also the activity of selected antioxidant enzymes, as well as GSH and MDA levels. Moreover, treatment probably affected vitamin C, but not vitamin D_3_ levels in horses. However, the initial fluctuations in the values of selected parameters remain unexplained. These variations could have been caused by individual adaptive responses to the subsequent light therapy sessions. These adaptive processes could have initially increased the antioxidant potential of cells. However, effects of longer exposure cannot be reliably predicted because an increase in the intensity or dose of the applied treatment can lead to cyclic fluctuations in the metabolic activity of mitochondria [74].

## Conclusion

Summing up, ten light therapy sessions performed with use of a solarium unit exerted a positive influence on the morphological parameters and the antioxidant potential of blood from recreational horses in winter. The presented results suggest that therapy may affect chosen blood indices and boost white blood system. This has mainly manifested by a simultaneous increase of MCV, HDW, MCVr, CHr and MPV indices as well as decrease in the basophil counts and MCHC, RDW and CHCMr indices in both groups of horses (p ≤ 0.05). Concurrently, a decline in reticulocytes in older and a rise in white blood cells and monocytes counts in younger individuals were observed. Furthermore, obtained results indicate that vitamin C and GSH were the main antioxidants in the blood plasma, whereas the activity of GPx and GR fluctuated subject to the demand for GSH. The maintenance of intracellular GSH reserves and the prevention of oxidative stress, measured by MDA levels, played the most important role in erythrocytes. Surprisingly, light therapy appears to give more prominent and stabilising effect in young horses. However, this finding could be partly attributed to individual adaptive responses to treatment or the greater responsiveness of the immune system of young animals.
